# Repair of fingertip defect with reverse digital artery island flap and repair of donor site with digital dorsal advancement flap

**DOI:** 10.3389/fsurg.2023.1127356

**Published:** 2023-04-12

**Authors:** Junwei Ma, Yunqi Ding, Lina Xu, Haibo Zou, Jinsheng Wu, Lin Shen, Changtai Xing, Yue Liu, Zehui Zhou, Jie Zhan

**Affiliations:** ^1^ Hand Surgery Department, Central Hospital Affiliated to Shenyang Medical Collage, Shenyang, China; ^2^ Department of Orthopedic Surgery, First Affiliated Hospital, China Medical University, Shenyang, China; ^3^ Department of Gynaecology, Shenyang Women's and Children's Hospital, Shenyang, China; ^4^ Operating Room, Central Hospital Affiliated to Shenyang Medical Collage, Shenyang, China; ^5^ Department of Anesthesiology, Central Hospital Affiliated to Shenyang Medical Collage, Shenyang, China

**Keywords:** fingertip skin defects, reverse digital artery island flap (RDAF), donor site renova, relaying flap, digital dorsal advance flap

## Abstract

**Objective:**

The reverse digital artery island flap (RDAF) is widely used in repairing fingertip skin defects based on its good appearance and practicability. However, the donor area of the flap needs skin grafting, which can lead to complications. This retrospective study explored the clinical application of digital dorsal advance flap (DDAF) in repairing the donor site of the reverse digital artery island flap.

**Method:**

From June 2019 to February 2022, 17 patients with a soft tissue defect of the finger had been restored with the reverse digital artery island flap, and at the same time, the donor area was repaired with digital dorsal advance flap (DDAF). The sensitivity, the active range of motion (ROM) and patient satisfaction were assessed after the operation.

**Results:**

All flaps survived completely without skin grafting with only one linear scar. The sensory and motor functions of all patients recovered well. Assessment based on the Michigan Hand Outcomes Questionnaire (MHQ) showed satisfactory functional recovery for all patients.

**Conclusions:**

Reconstruction using RDAF combined with DDAF represents an effective alternative for repairing fingertip skin defects.

## Introduction

Skin and soft tissue defects of the fingertip represent one of the most common traumatic injuries to the hand and are often challenging for hand surgeons and plastic surgeons since they can lead to irreversible hand sensation and movement impairment. In recent years, various homodigital and heterodigital flaps have been used to repair complicated fingertip defects because of the exposed bone of the fingertip and have shown several limitations. Different reconstructive treatment options have been designed. For instance, groin flaps have a reliable blood supply and are widely used in the reconstruction of limb function but rarely for the repair of finger defects due to the impact of long-term fixation on function, poor texture, thick flap and poor appearance (1). The cross-finger flap is a 2-staged procedure originally reported by Cronin in 1945 (2). Since then, this flap has mainly been used to repair the skin and soft tissue defects of the fingertip and pulp. This flap, taken from the dorsal side of the adjacent finger's middle phalanx, has a reliable blood supply and is relatively easy to operate. However, it is a 2-staged procedure, the finger adjacent to the skin graft is important, and the donor finger may become stiff. Gatewood et al. first reported the thenar flap in 1926 (3). Although the texture of the thenar skin is good, it is also a 2-staged procedure. It has been established that the donor area of the flap has a healthy muscle bed that is easily closed linearly, and skin grafts are rarely needed. However, prolonged immobilization may result in finger stiffness. In addition, if the nerve is damaged, normal sensation and movement of the thumb may be affected. The oblique triangular flap (4) and V-Y flap (5) are local advancement flaps of approximately 15 mm, only suitable for relatively mild skin injury.

The reverse digital artery island flap is widely used in repairing fingertip skin defects because of its good appearance and practicability (6). However, reconstruction using RDAF has been associated with some problems. When the conventional approach is adopted, the donor area cannot be sutured directly and needs to be repaired with a full-thickness graft instead. A second donor site is required to harvest a skin graft, which may affect metacarpophalangeal joint motion and additional morbidity related to a second donor site (7, 8).

To address this problem, Xianyao Tao et al. (9) designed a novel digital dorsal advance flap (DDAF) along the dorsal border of the reverse digital artery island flap as a relaying flap. The advantage of this modified approach is that the relaying flap can cover the donor defect and allow direct closure of the primary donor site without increasing damage to the second donor area. The appearance, function and sensation of the fingers experience a good recovery and the complications related to free skin grafting can be avoided.

Herein, we sought to demonstrate the clinical application of this modified operation by describing our clinical experience on 11 cases treated by this procedure.

## Patients and methods

From June 2019 to February 2022, 17 patients underwent the reverse digital artery island flap to repair fingertip defects at our hand surgery center, while the dorsal digital advancement flap was used to repair the donor area. The included patients had a mean age of 40.7 years (range, 19 to 65 years), with male predominance (*n* = 14, 82.3%) at the time of surgery. In most cases, the injury mechanism was sharp cuts (*n* = 9), followed by avulsion (*n* = 4) and crush injury (*n* = 4). 1 patient also underwent internal fixation of a finger fracture. Reverse digital artery island flaps ranging between 15 × 13 mm to 25 × 18 mm were used for fingertip reconstruction within 12 h of the initial trauma in all cases. In the meantime, the donor sites of all patients were repaired with digital dorsal advance flaps (Table 1). The study was approved by the Ethics Committee of Central Hospital Affiliated to Shenyang Medical Collage (202010). Signed informed consent was obtained from each patient.

**Table 1:** Patient demographics and surgical details.

Case	Age (years)	Sex	Affected hand, digit number	Cause	Defect site (cm)	Follo-up (months)
1	61	M	L,3	Sharp Cut	20 × 15	12
2	31	M	L,3	Avulsion	18 × 16	11
3	34	M	R,3	Sharp Cut	22 × 18	12
4	21	M	L,2	Sharp Cut	18 × 17	15
5	36	M	L,3	Sharp Cut	23 × 18	10
6	65	M	L,4	Crush	19 × 18	11
7	41	W	L,3	Avulsion	22 × 19	12
8	44	M	L,2	Crush	21 × 17	13
9	28	M	R,3	Sharp Cut	20 × 18	14
10	45	M	L,3	Sharp Cut	24 × 17	11
11	53	M	R,2	Sharp Cut	20 × 17	9
12	36	M	R,2	Crush	21 × 18	8
13	19	W	R,2	Sharp Cut	25 × 18	9
14	35	M	L,3	Sharp Cut	20 × 18	15
15	55	W	R,2	Avulsion	21 × 17	18
16	43	M	R,4	Avulsion	15 × 13	17
17	44	M	R,2	Crush	16 × 14	16

L, left; R, right; M, male; F, female.

## Surgical technique

In all cases, an axillary block of the affected side was conducted for anesthesia, and a tourniquet was inflated around the upper arm. During debridement, if more than two-thirds of the nail bed was missing, the nail bed and onychostroma were completely removed to avoid postoperative pain and the sharp phalangeal trochanter was trimmed.

**Figure 1:**
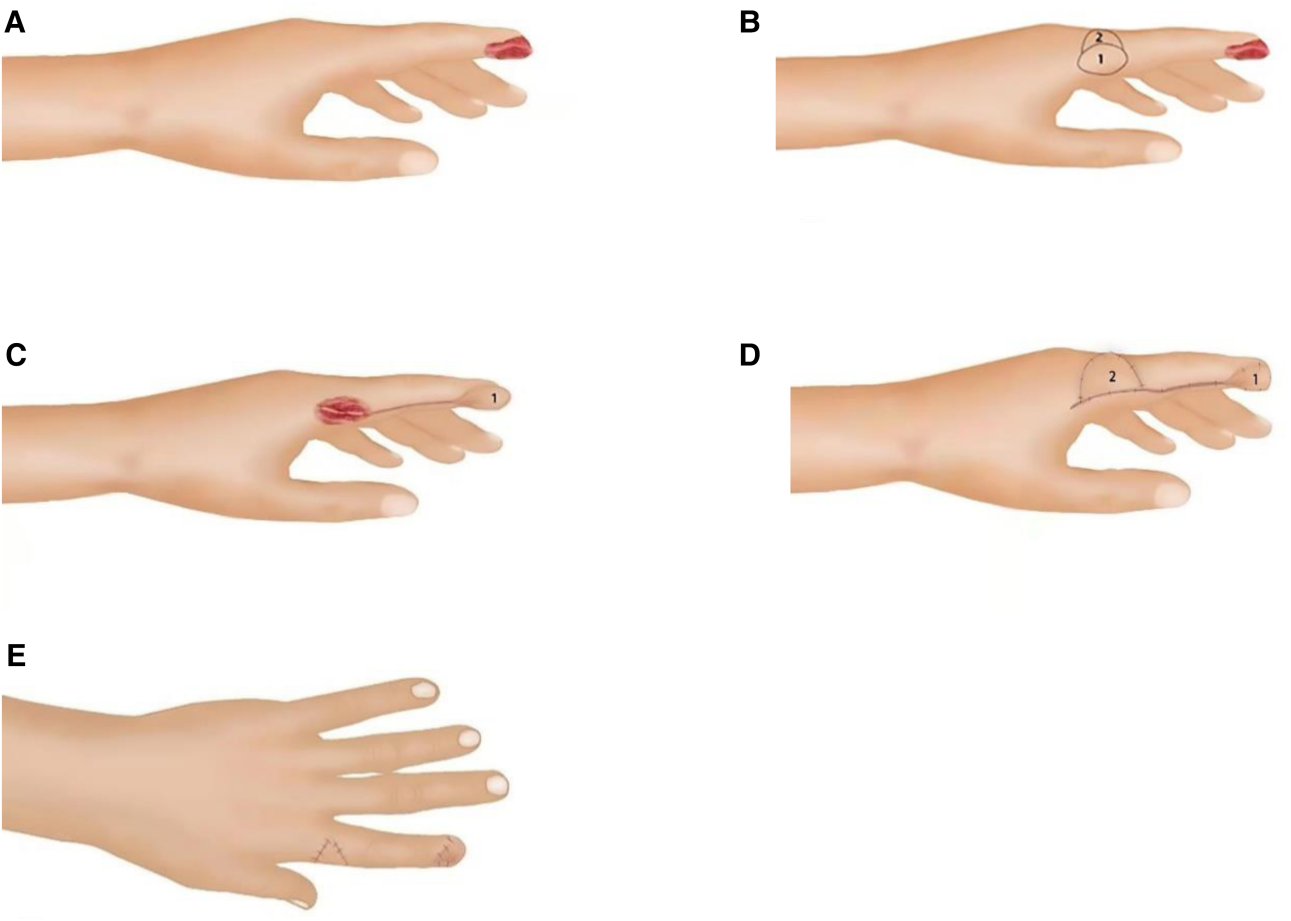
Schematic diagram of operation. (**A**) Fingertip skin defect with bone exposure; (**B**) According to the size and shape of the skin defect, the reverse digital artery island flap and dorsal digital advancement flap were designed at the proximal finger; (**C**) The reverse digital artery island flap was harvested to cover the fingertip skin defect maintaining the continuity of the proper digital nerve. (**D,E**) The donor area of the reverse digital artery island flap is covered by the reverse digital artery island flap.

According to the general principles of flap design, the flap size was 20% larger than the skin defect. The designed flap with digital artery was taken on the lateral side of the trauma proximal phalanx. The flap shape was designed similarly to the skin defect. As the axial blood vessel of the flap, the digital artery must be included in the flap. The pivotal point was generally designed at the level of the middle phalanx neck, which is the position of the perforating branch of the opposite digital artery, and cannot exceed the distal interphalangeal joint. The vascular pedicle of the flap coursed with the digital artery. The above elements were designed and marked on the injured finger before the operation (Figures 1A,B). The nerve and vascular bundle were carefully separated at the finger's base. Based on our experience, the digital artery was included in the flap while maintaining the continuity of the digital nerve (Figure 1C). The digital artery was sectioned and ligated at the base of the finger. A 0.5 to 0.6 cm fascia pedicle containing veins and capillaries was retained around the vascular pedicle, making sure not to separate the digital artery from the rest to increase arterial blood supply and flap venous return (10). The vascular pedicle of the flap was raised to the pivotal point. We rotated the flap 180° at the pivot point to cover the fingertip defect (Figure 1C). The proper digital nerve was not broken and need not to be repaired by suturing.

When the conventional surgical approach was adopted, the skin graft was often needed in the donor area. We designed and cut the triangular digital dorsal advanced flap with the dorsal border of the RDAF as the base. Two incisions were made, and the subcutaneous layer was carefully dissected to protect the nerves and blood vessels. The digital dorsal advance flap was advanced and covered the donor area of the reverse digital artery island flap, which was used as a relaying flap (Figures 1D,E). The remaining Y-shaped incision on the dorsal of the finger was directly drawn and sutured. A postoperative drainage strip was placed to prevent hematoma formation from affecting the flap's blood supply and pulled out on the next day.The hand was elevated for 1 week postoperatively.

## Evaluation of outcomes

At the final follow-up, we checked the sensory function recovery of flaps through a two-point discrimination (2-PD) test (11).

The first RDAF for the primary defect of the finger and the relaying flap were evaluated, respectively, and compared with the contralateral hand. The active range of motion (ROM) was measured using a standard goniometer. The total active ROM was calculated as the sum of the ROM of the interphalangeal joint of the finger and the sum of the ROM of the metacarpophalangeal joints of the finger.

We also evaluated all patients' limitations and satisfaction in daily life with The Michigan Hand Outcomes Questionnaire (MHQ) (12), an evaluation system that can effectively evaluate patients' subjective limitations and satisfaction. It includes 37 items and 6 subscales: pain, overall hand function, activities of daily living (ADL), work performance, aesthetics and patient satisfaction with hand function. The raw score for each of the 6 scales is converted to a score ranging from 0 to 100 using a general algorithm. Higher scores were associated with more pain on the pain scale and better hand performance on the other scales.

## Results

**Figure 2:**
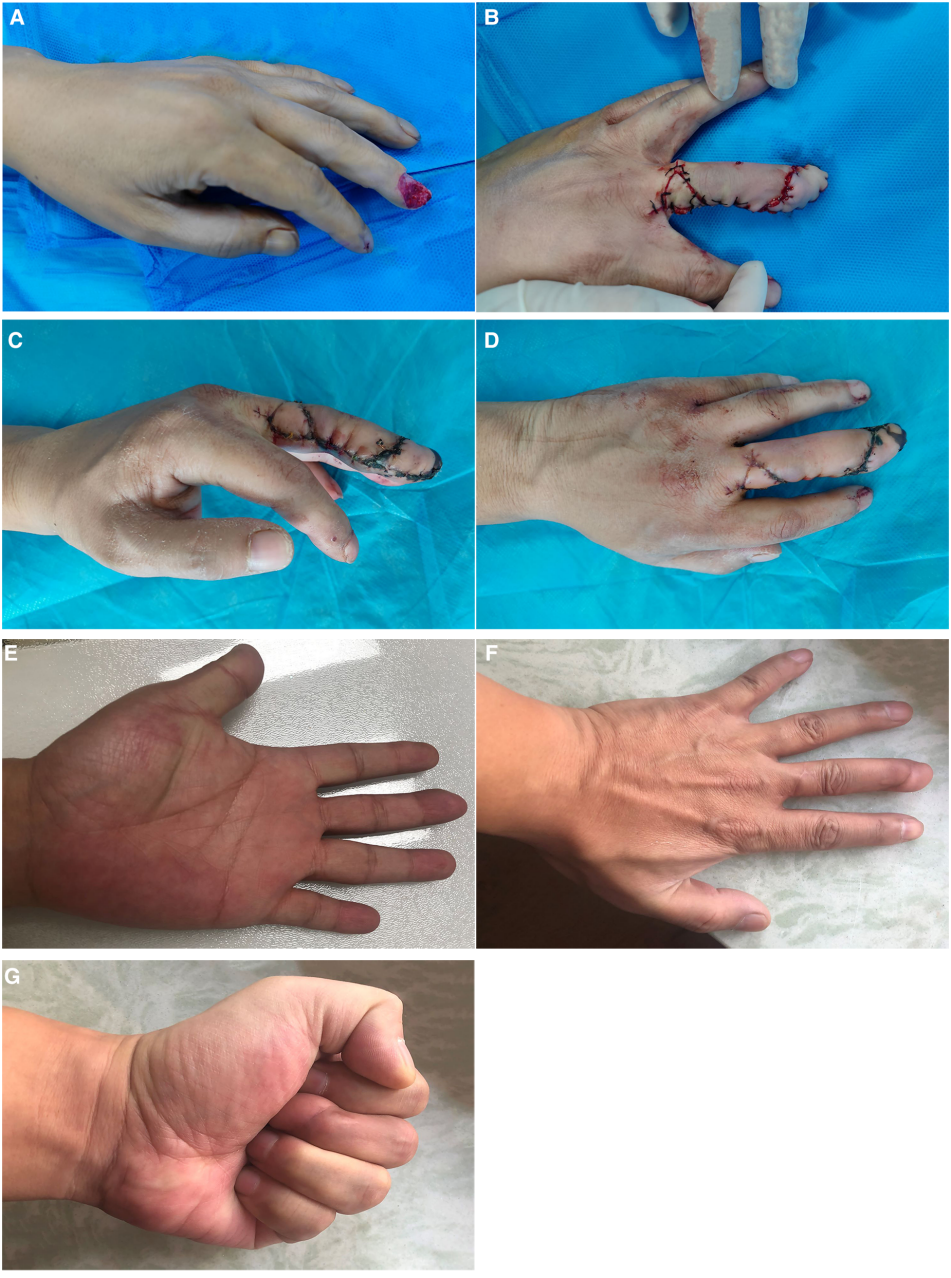
Case: 10 (**A**) Skin and soft tissue defect on the fingertip of the left middle finger. (**B**) The defect of fingertip was repaired by digital artery island flap, and a digital dorsal advancement flap was used to cover the donor site. (**C,D**) The incision healed well and the flap survived completely after two weeks of operation. (**E–G**) A 11-month follow-up after the operation.

All flaps survived completely without surgical complications, and no skin graft operations was required. The operation time ranged from 45 min to 65 min, with an average of 52 min. The follow-up was conducted for an average of 12 months (8–22 months).The mean static 2-PD in the reverse digital artery island flap was 4.8 mm (3.2–5.8 mm). The mean total active ROM of the finger was very satisfactory, which reached 93.4% compared to the opposite side. The postoperative assessment of sensation and active ROM were shown in Table 2. The postoperative assessment for subjective evaluation is summarized in Table 3. According to the results of MHQ, the overall hand function, activities of daily living, work performance, pain, aesthetics, and satisfaction scores were 94.5, 93, 96.5, 3.7, 89.5, and 91.5, respectively (Figure 2, cases 10).

**Table 2:** Postoperative assessment of sensation and active range of motion (ROM).

Case	2-PD Test (mm)	Numbness	Tinel's sign	%TAM(%)
1	5.1	Y	Y	85.5
2	5.4	N	N	95.7
3	5.8	Y	Y	86.5
4	5.6	N	N	96.6
5	4.1	N	N	96.5
6	4.0	N	N	95.5
7	3.9	N	N	94.5
8	5.5	N	N	95.5
9	5.1	N	N	94
10	3.9	N	N	94.5
11	6.3	N	N	93.4
12	4.3	N	N	93.5
13	4.1	Y	Y	89
14	4.2	N	N	92.4
15	3.2	N	N	93
16	5.2	N	N	96
17	5.3	N	N	95

**Table 3:** Postoperative assessment of subjective evaluation (five domains of the MHQ).

Case	Overall hand function	Activities of daily living	Work performance	Pain	Aesthetics	Satisfaction
1	90	92	93	4	89.5	89
2	96	97	96	3.5	88	90
3	96	94	97	4.5	95	91.5
4	85	87	88	5	86	86
5	93.5	92	96.5	3.5	89	91
6	85.7	89.5	95.5	4.4	89.5	91.5
7	85	88.5	92.5	5.1	86.5	90.5
8	88.5	91.5	90	4.1	89.5	91.5
9	86	91	94	3.6	90	89
10	94.5	93	96.5	3.7	89.5	91.5
11	87.5	90.5	91	5.5	88.5	85.5
12	92	95	94	3.5	90	91.5
13	94	93	95	4	93.5	91
14	92	95.5	95.5	3	90	90.5
15	87.5	92.5	92.5	3.5	90	87.5
16	92	94	93.5	4.5	94.5	91.5
17	93	96	95	3	92.5	91.5

MHQ, the michigan hand outcomes questionnaire.

## Discussion

Restoring the original length is important for functional recovery during the repair of a traumatic finger injury. Over the years, many methods have been reported to repair fingertip defects; one of the most effective methods is the digital artery reverse island flap, which is perfused by the perforator vessel between two digital arteries (13). It is widely acknowledged that there are many perforator vessels along both sides of the finger, and in most cases, vessels are usually operated at the middle phalangeal level.

The advantages of the digital artery reverse island flap include stable and reliable blood supply, thin skin flap and good texture. Y. C. Sun et al. (14) also found that the fingers repaired by RDAF exhibited good cold resistance, and the sensory function of the flap also recovered well. However, much controversy surrounds the restoration of sensation. Lai et al. substantiated that good sensory function was restored by suturing the dorsal branch of the digital nerve (15). However, Han et al. reported that sensory function recovery was not affected by suturing of the nerve (16). Sapp et al. (17) reported that the reason was that the peripheral nerve of the flap could quickly dominate the flap similar to the nerve recovery when the fingertips are replanted without connecting the finger nerve (18). So according to our experience, we haven't done any nerve anastomosis, and the nerve function still recovered well (Table 2). This phenomenon may be attributed to the fact that the digital artery reverse island flap is small and can be more easily re-innervated by the peripheral nerves of the flap. Besides, the blood supply of the flap is reliable, and the texture of the flap is similar to that of the missing skin.

Ali Güleç et al. (19) reported that Allen's test and a Doppler examination should be performed before surgery to ensure the existence of communicating branches between digital arteries. Based on our experience, these are not necessary because of the reliable blood supply of the flap and the presence of fascia tissue in the vascular pedicle.

Therefore, our results corroborate that repairing fingertip defects with RDAF yields excellent results. However, the donor site of the flap often needs skin grafting. This may result in problems such as hyperpigmentation and hypertrophic scarring (20), which may influence the restoration of sensations. Moreover, the joint motion may be affected by skin contractures, which lead to decreased range of motion of the joint.

Many authors tried to use a local transfer flap instead of a skin graft to repair the donor area of the flap and achieved good results. For example, Matthew A et al. (21) found that Simple syndactyly reconstruction with a dorsal metacarpal advancement flap may lead to fewer complications than skin graft procedures.

To avoid the external depression and scar contracture of the donor area of the RDAF by skin grafting, some authors used the dorsal metacarpal artery perforator flap to repair the donor area (22). Although the dorsal metacarpal artery was protected, the donor area of the flap was severely damaged, and the dorsal hand wound was longer, affecting the appearance. Zhenglin Chi et al. (23) used the first dorsal metacarpal artery flap to repair the thumb skin defect and the second dorsal metacarpal artery flap to repair the donor area, while the donor area of the second dorsal metacarpal artery flap was directly sutured.

Along with the digital artery island flap, there was also some subcutaneous tissue transferred to the fingertip, Accordingly, the donor area was covered by a smaller flap, which just makes up for the limited range of the dorsal digital advancement flap. The advantages of the dorsal digital advance flap, include reliable blood supply, high survival rate, good texture of the flap, and negligible damage, which are exactly what the donor area needs. Moreover, metacarpophalangeal joint movement is better than skin grafting.

Based on our experience, using a dorsal advancing flap as a relay flap can yield a good appearance and function, the sensory function of the digital dorsal advance flaps is significantly better than skin grafting and approached the the opposite finger (Table 1). Most importantly, the flap has a very high survival rate, and the secondary defect is easily closed linearly because of the continuity of the subcutaneous tissue. Functional assessment measured by the Michigan Hand Outcomes Questionnaire provides the theoretical basis for wider clinical implementation of this technique (Table 3).

However, several limitations were found in this study, including the relatively small sample size. In addition, the technique exhibits limited value when applied to large skin defects. This retrospective study has its limitations, More prospective, randomized, and blinded studies are warranted for more reliable findings of the efficacy of RDAF combined with DDAF. In recent years, the perforator flap has gradually been widely popularized and applied. Next, we will also apply the perforator flap technology to repair the fingertip defect through reverse digital artery island flap, which will definitely make the flap thinner, more beautiful and less damage in the donor area, Which would benefit more patients.

## Conclusion

Fingertip reconstruction using RDAF combined with DDAF is a novel approach with less morbidity, better functions, and aesthetics. This improved surgical method has huge prospects for repairing fingertip skin defects.

## Data Availability

The original contributions presented in the study are included in the article/Supplementary Material, further inquiries can be directed to the corresponding authors.
