# Supplementary figures and images for: Repair of fingertip defect with reverse digital artery island flap and repair of donor site with digital dorsal advancement flap
Source: Front Surg. 2023 Apr 12;10:1127356. doi: 10.3389/fsurg.2023.1127356 (PMC10130516; doi:10.3389/fsurg.2023.1127356)

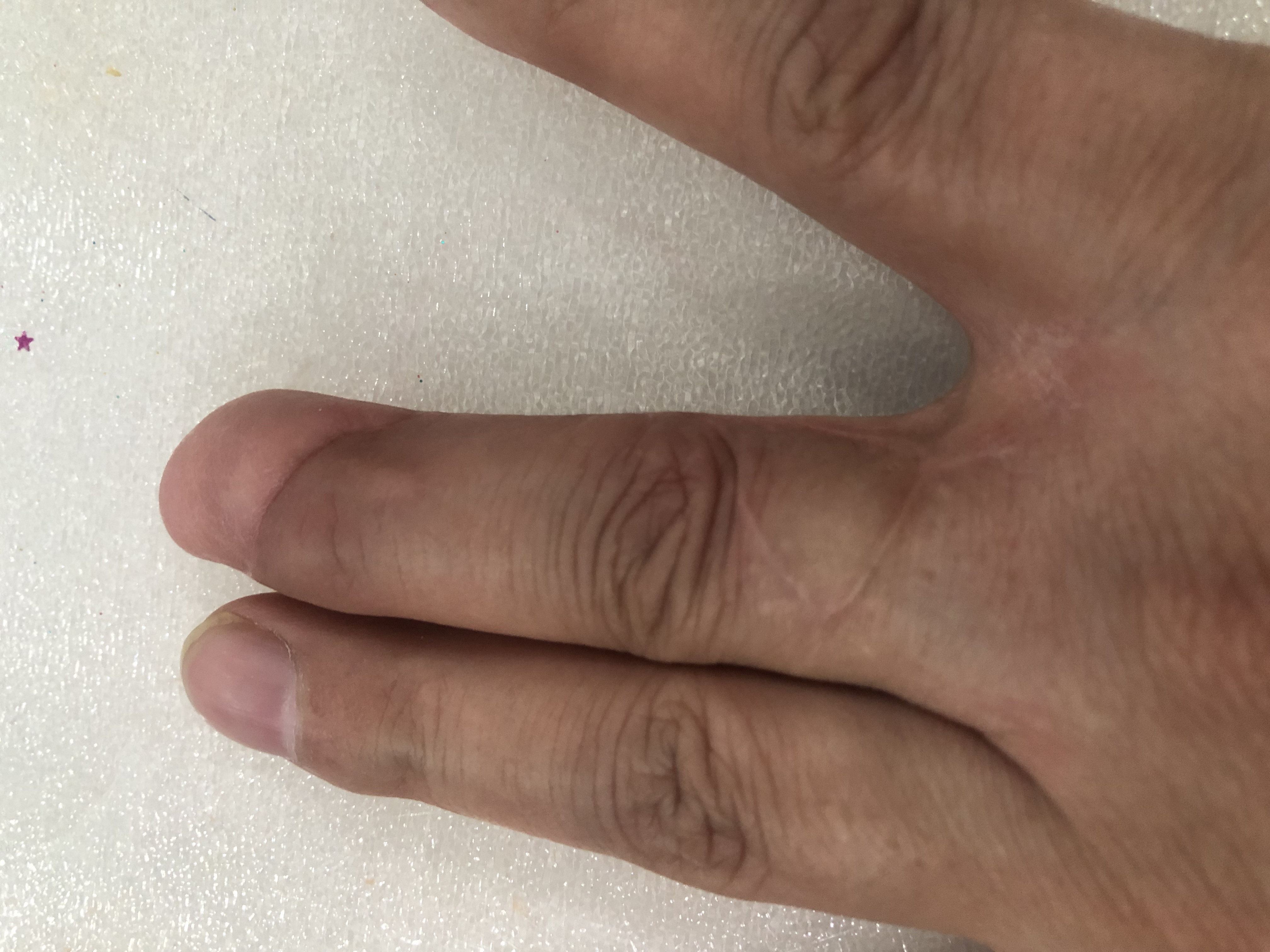

Supplement: Supplementary file 3 [file Image1.jpeg]

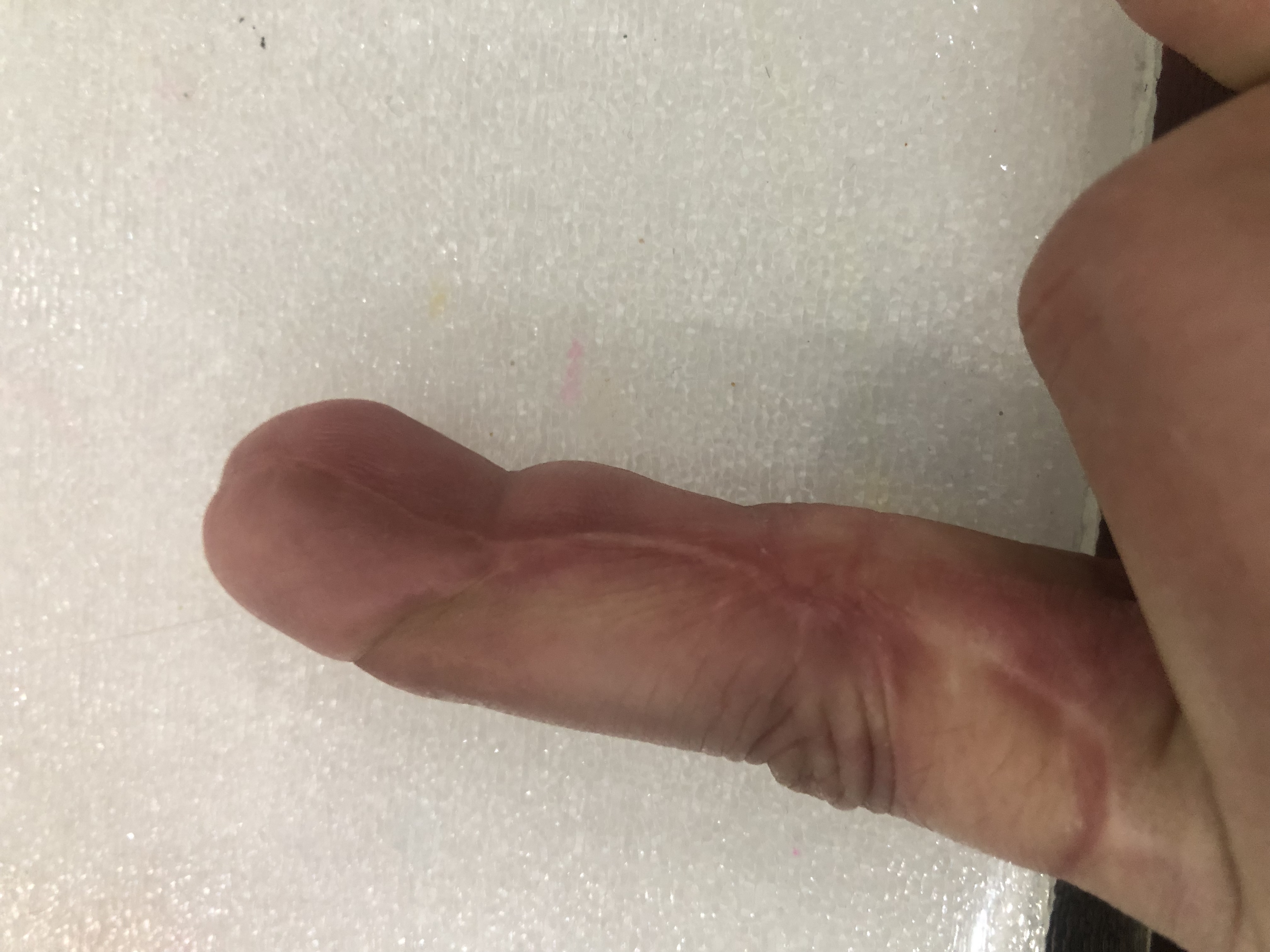

Supplement: Supplementary file 4 [file Image2.jpeg]

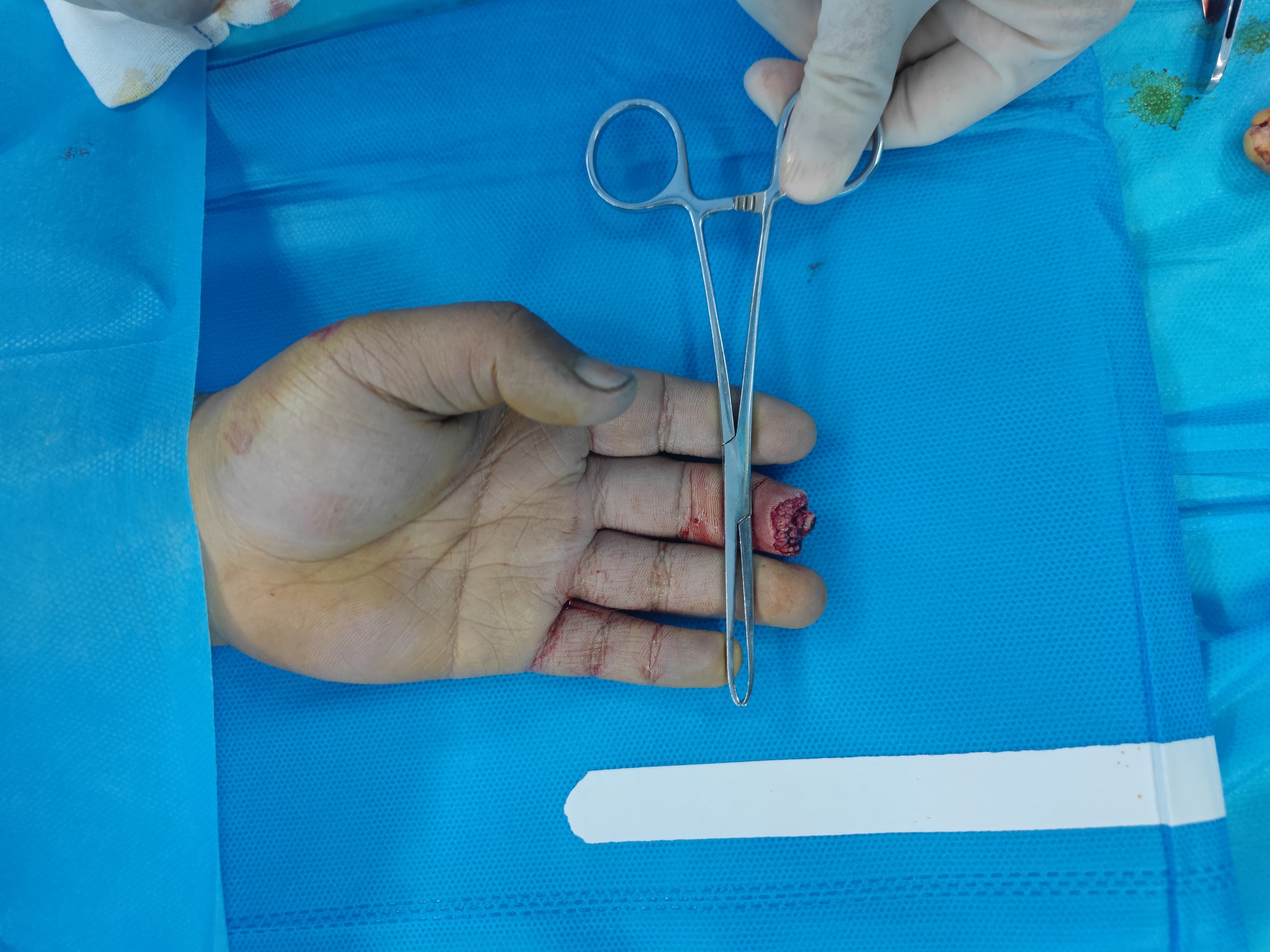

Supplement: Supplementary file 5 [file Image3.jpeg]

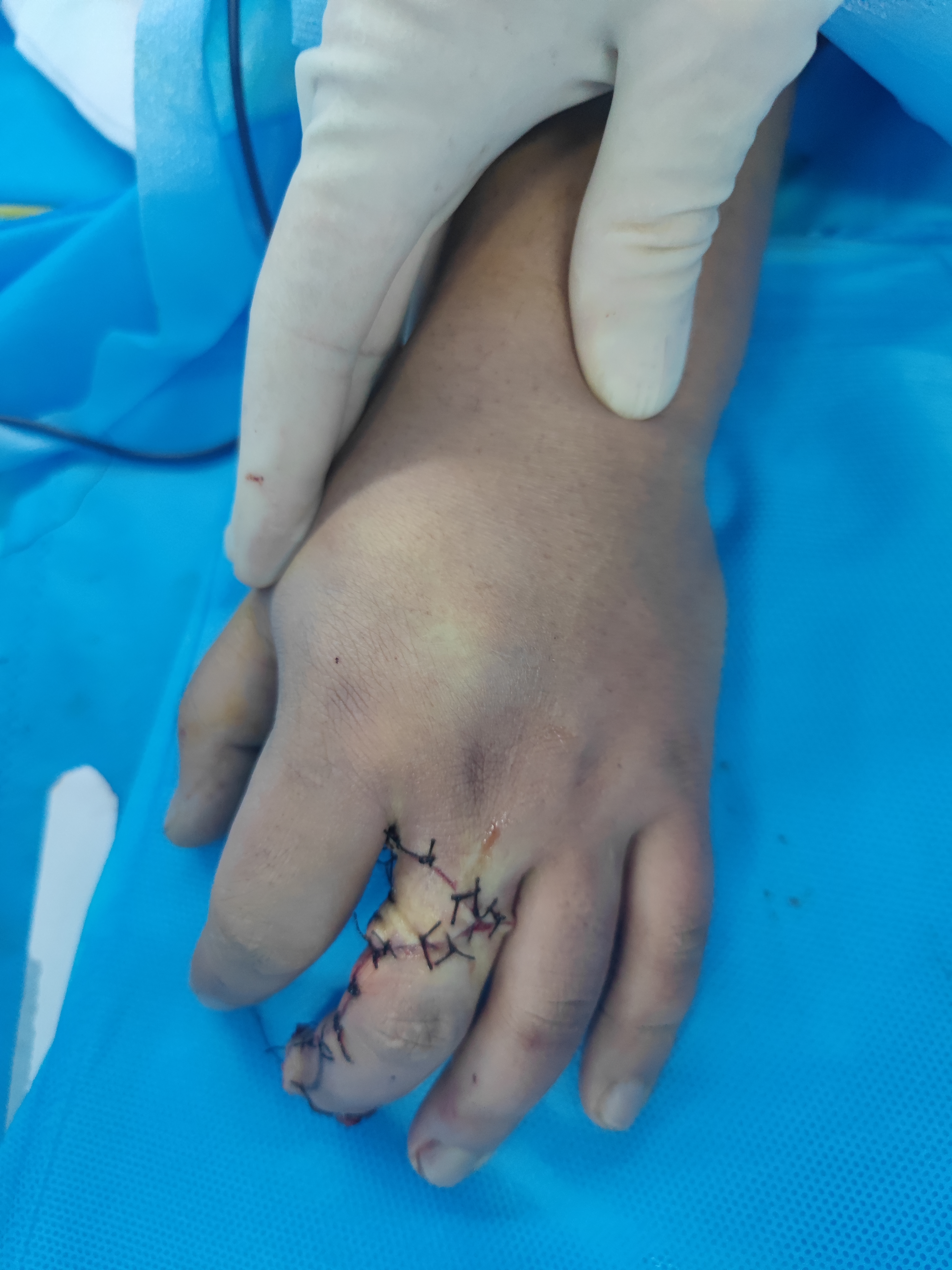

Supplement: Supplementary file 6 [file Image4.jpeg]

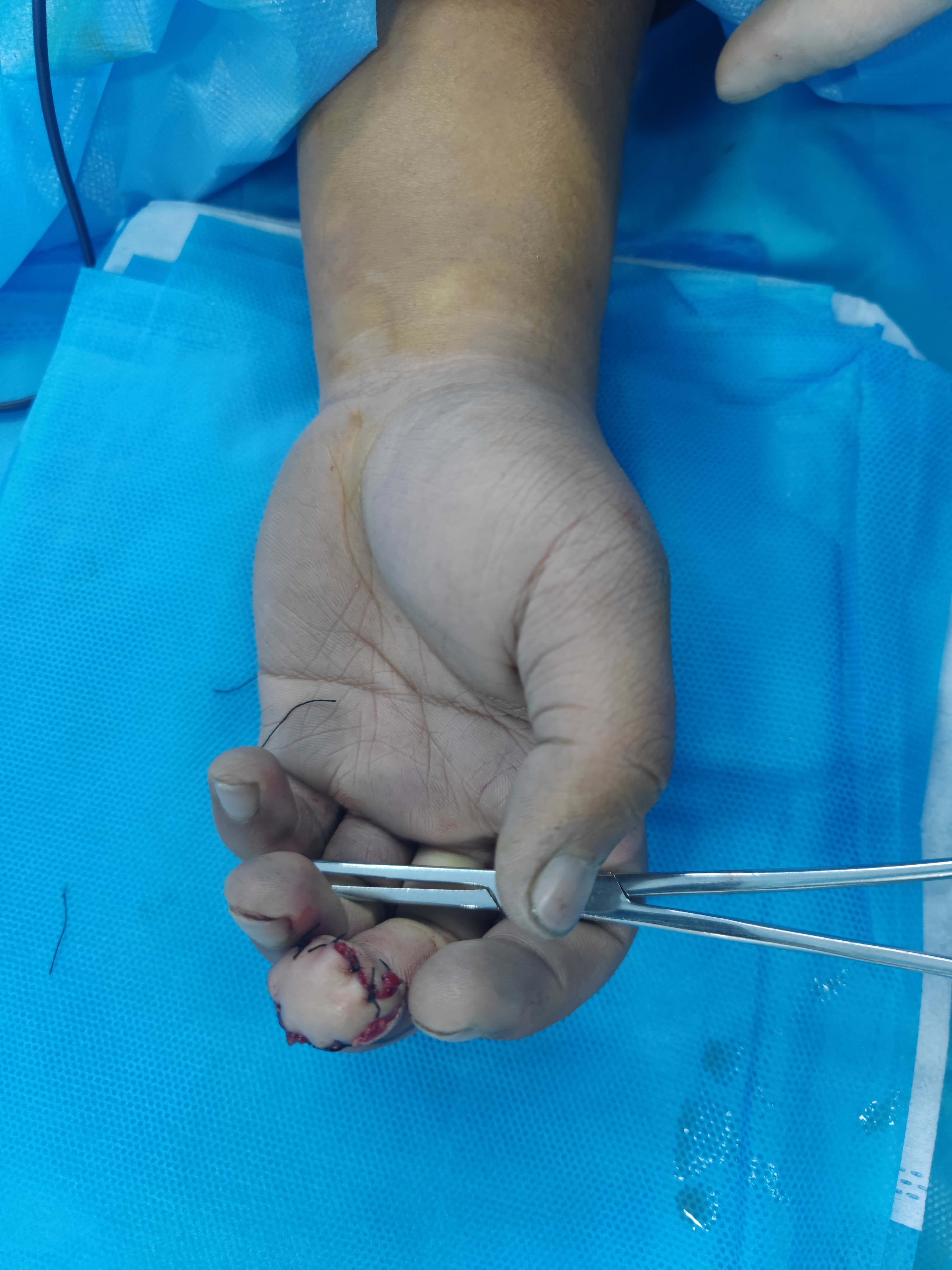

Supplement: Supplementary file 7 [file Image5.jpeg]
